# SARS-CoV-2 Antibodies in Response to COVID-19 Vaccination in Underserved Racial/Ethnic Minority People Living with HIV

**DOI:** 10.3390/vaccines13050517

**Published:** 2025-05-13

**Authors:** Yongjun Huang, Haley R. Fonseca, Leonardo Acuna, Wensong Wu, Xuexia Wang, Samantha Gonzales, Manuel Barbieri, David R. Brown, Marianna K. Baum

**Affiliations:** 1 Robert Stempel College of Public Health and Social Work, Florida International University, Miami, FL 33199, USA; yongjunhuang1001@gmail.com (Y.H.); hmart101@fiu.edu (H.R.F.); lacun005@fiu.edu (L.A.); xuexwang@fiu.edu (X.W.); samagonz@fiu.edu (S.G.); 2 Department of Mathematics and Statistics, College of Arts, Sciences & Education, Florida International University, Miami, FL 33199, USA; wenswu@fiu.edu; 3 Department of Biological Sciences, College of Arts, Sciences & Education, Florida International University, Miami, FL 33199, USA; barbieri@fiu.edu; 4 Herbert Wertheim College of Medicine, Florida International University, Miami, FL 33199, USA; drbrown@fiu.edu

**Keywords:** HIV, COVID-19, SARS-CoV-2, vaccine, vulnerable, underserved, antibodies, viral load, CD4 cell count, immune response

## Abstract

**Background**: Understanding immune response is essential for preparing for public health crises. COVID-19 vaccination provides robust immunity against SARS-CoV-2, but immunocompromised populations may have weaker immune responses. We assessed SARS-CoV-2 spike (trimer) total IgG/IgM/IgA (total Ig) to investigate immune response to COVID-19 vaccination in people living with HIV (PLWH), considering CD4^+^ T cell count, viral load, substance use, and comorbidities. **Methods**: This cross-sectional study was conducted in Miami, Florida, between May 2021 and December 2021 as part of the NIH Rapid Acceleration of Diagnostics-Underserved Populations (RADx-UP) initiative (3U01DA040381-05S1) and the Miami Adult Studies on HIV (MASH) cohort (U01DA040381). Blood samples were collected and SARS-CoV-2 spike (trimer) total Ig was quantified. HIV serostatus, viral load, CD4^+^ T cell count, and COVID-19 vaccinations were abstracted from medical records. Substance use (tobacco, alcohol, and drug use [marijuana, cocaine, heroin, fentanyl, methamphetamine, amphetamine, hallucinogens, ecstasy, or misuse of prescription drugs]), and comorbidities (hypertension, diabetes, autoimmune disease, obesity, chronic kidney disease, and substance use disorders) were assessed via validated questionnaires. Drug use was confirmed via urine toxicology. Multivariable linear regression was conducted. **Results**: Median age (*n* = 1317) was 57.8 years, 49.8% were male, 50% were Black non-Hispanic, 66.2% had received ≥1 dose of a COVID-19 vaccine, and 29.6% were PLWH (71.3% virally suppressed and median CD4^+^ T cell count > 500 cells/µL). PLWH, compared to people without HIV, were more likely to have received ≥1 dose of a COVID-19 vaccine (76.2% vs. 62.0%, *p* < 0.001) and present with substance use (77.2% vs. 42.9%, *p* < 0.001) and comorbidities (72.8% vs. 48.2%, *p* < 0.001). Vaccinated PLWH, compared to unvaccinated PLWH, had higher CD4^+^ T cell counts (577.5 vs. 517.5, *p* = 0.011) and were more likely to be virally suppressed (76.4% vs. 54.8%, *p* < 0.001). A lower CD4^+^ T cell count (<200 vs. ≥500, β = −0.400, *p* = 0.033) and higher HIV viral load (≥200–<5000 vs. <200, β = −0.275, *p* < 0.001) were associated with lower spike (trimer) total Ig titers, indicating a diminished response to COVID-19 vaccination. **Conclusions**: A lower CD4^+^ T cell count and higher HIV viremia were linked to reduced SARS-CoV-2 immunogenicity in racial/ethnic minority PLWH, a population underrepresented in vaccine clinical trials. HIV care providers should target efforts to maintain viral suppression to avoid diminished responses to COVID-19 vaccination.

## 1. Introduction

People living with HIV (PLWH) have a greater risk of severe acute respiratory syndrome coronavirus 2 (SARS-CoV-2) infection and the development of severe Coronavirus disease-2019 (COVID-19) [1,2,3]. The degree of risk is, in part, dependent on the management of HIV viremia, which negatively impacts host immune response [3,4]. A successful immune response results from the coordinated efforts of white blood cells such as B cells, T cells, and antibodies (immunoglobulins [Ig]) [5,6]. These components work together in a highly orchestrated manner to recognize, neutralize, and eliminate pathogens, and provide long-term protection through the generation of memory B cells [5,6]. In PLWH, these elements, particularly CD4^+^ T cells, are diminished [7,8]. The depletion of CD4^+^ T cells weakens the ability to mount an effective immune response, making individuals more susceptible to opportunistic infections [8]. A progressive decline in CD4^+^ T cell count is a hallmark of untreated HIV infection and is a key factor in the progression to acquired immunodeficiency syndrome (AIDS) [8].

Antiretroviral therapy (ART) suppresses HIV replication and restores CD4^+^ T cell count in PLWH [8]. Despite advancements in ART, the immune system of PLWH may not experience complete restoration and is characterized by residual inflammation and immune system dysregulation [9], culminating in a diminished response to immunization [10]. Seroconversion rates following vaccination for various diseases (e.g., pneumococcal disease, hepatitis B, measles, mumps, rubella [MMR], and yellow fever) in PLWH are lower compared to uninfected controls, and PLWH experience a more rapid decline in immunity [11,12,13,14,15]. Thus, the Centers for Disease Control and Prevention (CDC) developed vaccination guidelines specific to PLWH for a range of diseases, which include additional doses and more frequent boosters [16].

COVID-19 vaccination is effective in providing robust immunity against SARS-CoV-2, reducing severe illness in immunocompetent recipients [17,18,19,20,21,22]. COVID-19 vaccination has been reported to be just as effective in PLWH on ART with normal CD4^+^ T cell counts and a suppressed viral load, compared to uninfected controls, in countries outside of the US [23,24,25,26] and in small US samples [27]. However, other studies reported that PLWH present with a diminished immune response compared to uninfected controls [23], particularly in PLWH with lower CD4^+^ T cell counts [24], suggesting vaccine response varies based on immunosuppression level. However, COVID-19 vaccine trials included only small samples of virally suppressed PLWH on ART with normal CD4^+^ T cell counts [25], and did not consistently publish information on immunogenicity in PLWH [23,28]. Additionally, vaccine research, including COVID-19 vaccines, underrepresent racial/ethnic minorities, with most studies overrepresenting White non-Hispanic populations [23,25,26].

In addition to HIV, cardiometabolic comorbidities and substance abuse negatively impact immune function [29,30,31,32,33,34]. This suggests that PLWH and comorbidities may face a dual disadvantage, particularly in matters requiring carefully orchestrated immune functions, such as responding to vaccination. In fact, many comorbidities have already been associated with fewer neutralizing antibody titers against SARS-CoV-2 and poorer prognosis upon infection [35,36,37]. We aimed to investigate COVID-19 vaccine response in underserved, racial/ethnic minority PLWH by examining neutralizing antibody titers following two-dose SARS-CoV-2 vaccination and examining factors associated with SARS-CoV-2 immunogenicity, considering CD4^+^ T cell count, HIV viral load, comorbidities, and substance abuse.

## 2. Materials and Methods

### 2.1. Study Population

This cross-sectional study was conducted as part of the National Institutes of Health (NIH) Rapid Acceleration of Diagnostics-Underserved Populations (RADx-UP) initiative: a consortium of 144 projects studying COVID-19 testing patterns in underserved communities [38]. We analyzed data from an individual RADx-UP Phase I project site (3U01DA040381-05S1) located in an underserved urban sector of Miami, Florida; data for this analysis were collected between May 2021 and December 2021. Recruitment included participants in the Miami Adult Studies on HIV (MASH) cohort (U01DA040381) funded by the National Institutes on Drug Abuse (NIDA), which follows 1500 underserved Black and Hispanic adults living with and without HIV and high rates of comorbidities and substance use [39]. Detailed information about the methodology of this RADx-UP project site is provided elsewhere [40]. Briefly, the inclusion criteria for this RADx-UP project were being ≥18 years of age, and exclusion criteria included pregnancy. Eligible participants completed a survey that included validated measures of substance use, comorbidities, and health disparities, among other measures. At our research clinic, participants then underwent a blood draw and nasopharyngeal swab, which was tested for SARS-CoV-2 with real-time reverse transcription-polymerase chain reaction (rt-PCR). For this analysis, we included RADx-UP participants with complete data on HIV serostatus, COVID-19 vaccination, and SAR-CoV-2 antibodies. For the main analysis, since the sample size of participants who received only one dose of a COVID-19 vaccine was small (*n* = 33), we included PLWH who received two doses of a COVID-19 vaccine and excluded PLWH who received mixed vaccines (*n* = 6), PLWH who received two doses of the Ad26.COV2.S or BIBP-CorV inactivated COVID-19 vaccine (*n* = 4) [41,42], PLWH who underwent serology collection less than 14 days or more than 180 days after the second COVID-19 vaccine dose (*n* = 47) [43,44], nucleocapsid IgG seropositive cases (IgG ≥ 20 U/mL; indicates a prior natural SARS-CoV-2 infection; *n* = 22) [23,24], and those missing CD4^+^ T cell count or HIV viral load data (*n* = 22) (Supplementary Figure S1). The protocol for this study was approved by the Institutional Review Board at Florida International University; all participants provided informed consent to participate and to the release of their medical records.

### 2.2. Exposures: HIV Serostatus and COVID-19 Vaccination Status

HIV serostatus, HIV viral load, CD4^+^ T cell counts, ART, and COVID-19 vaccination information, including vaccination doses, dates, and brands, were abstracted from medical records.

### 2.3. Outcomes: SARS-CoV-2 Antibodies

Participants underwent a blood draw at our clinic; samples were drawn by a trained phlebotomist or registered nurse. Serum was isolated from whole blood via centrifugation. Two commercial assays were then used to test the serum samples for (1) SARS-CoV-2 nucleocapsid immunoglobulin G (IgG) to assess prior natural infection and (2) spike (trimer) total IgG/IgM/IgA (total Ig) to assess immune response to COVID-19 vaccination. The EDI™ COVID-19 Nucleocapsid IgG Quantitative ELISA Kit (Epitope Diagnostics, Inc., San Diego, CA, USA, Cat. # KTR-1034) was used to quantify the full-length SARS-CoV-2 nucleocapsid IgG. The Human SARS-CoV-2 Spike (trimer) Ig Total ELISA Kit (Invitrogen, Inc., Waltham, MA, USA, Cat. # BMS2323) was used to quantify spike (trimer) total IgG/IgM/IgA (total Ig).

### 2.4. Covariates: Sociodemographic Characteristics, Substance Use, and Comorbidities

Sociodemographic characteristics, including sex assigned at birth and race/ethnicity, were self-reported via standardized measures from RADx-UP common data elements (CDEs), which included items from the NIH CDE Repository, Disaster Research Response guidelines, and the PhenX Toolkit [45]. Age was confirmed via government-issued identification. Substance use, including tobacco (past 30 days), drug (past 12 months), and alcohol use (typical drinking habits), was determined via RADx-UP CDEs, which utilized the PhenX Toolkit [45]. Drug use included marijuana, cocaine/crack, heroin, fentanyl, methamphetamines, hallucinogens, ecstasy, or misuse of prescription drugs. For participants simultaneously enrolled in the MASH cohort, marijuana, cocaine, opioids, methamphetamine, amphetamine, and fentanyl use were verified via urine toxicology. Herein, drug use is defined as the use of marijuana, cocaine/crack, heroin, fentanyl, methamphetamines, amphetamines, hallucinogens, or ecstasy, or the misuse of prescription drugs, while substance use denotes the use of alcohol, tobacco, and/or drugs. Hazardous alcohol use was defined as >14 drinks/week for men (or >4 drinks/occasion), >7 drinks/week for women (or >3 drinks/occasion), and >7 drinks/week for adults ≥65 years [46]. Chronic conditions were assessed with the Johns Hopkins University C4-Ward Module Five: Comorbidities and Care Engagement, and included hypertension, diabetes, autoimmune diseases, chronic kidney disease (CKD), and substance use disorders [47]. Height and weight were measured to obtain body mass index (BMI).

### 2.5. Statistical Analyses

Descriptive statistics are presented as counts (percent, %) for categorical variables and median (interquartile range) for continuous variables. For categorical variables, between-group differences were tested using the chi-square test; Fisher’s exact test was utilized in cases of small cell counts. Due to the non-normality of continuous variables, the Wilcoxon rank-sum test was used to assess between-group differences. Boxplots were also generated and examined to compare median values of spike (trimer) total Ig by CD4^+^ T cell count and HIV viral load category. The main exposures of interest were HIV serostatus, HIV viral load, CD4^+^ T cell count, and COVID-19 vaccination. The primary outcome was spike (trimer) total Ig (indicates immune response to COVID-19 vaccination). The main linear regression analysis consisted of PLWH who received two doses of a COVID-19 vaccine, further divided by CD4^+^ T cell counts: <200, ≥200–<500, and ≥500 cells/µL [24,28]. Box–Cox transformations of the continuous outcome variable (spike [trimer] total Ig) were performed to bring residuals closer to normal distributions [23,24]. We also explored different BMI cut-offs in an exploratory analysis to find a suitable cut-off value with a potential association with SARS-CoV-2 spike (trimer) total Ig titer in this unique sample for use as a covariate representing BMI in multivariable models. A reduced model was developed with forward and backward stepwise variable selection. Missing data were treated as missing at random (MAR) and excluded from primary multivariable linear regression analysis [48]. A supplementary sensitivity analysis in which we compared the findings from the overall dataset of PLWH who received two doses of a COVID-19 vaccine with the subset used in the regression model was also conducted to evaluate the assumption that the data were in fact, MAR. Results were considered statistically significant at two-tailed *p* < 0.05. All statistical analyses were performed with R version 4.0.3.

## 3. Results

The sample (*n* = 1317) had a median age of 57.8 years (50.7–63.4); 49.8% were male, 50% were Black, non-Hispanic, 29.6% were living with HIV, 66.2% received at least one dose of a COVID-19 vaccine, and 75.5% of vaccinated participants underwent serology collection 14–179 days after their second vaccination dose (Table 1). Most PLWH were on ART (93.3%), virally suppressed (viral load < 200 copies/mL; 71.3%), and had a median CD4^+^ T cell count > 500 cells. PLWH, compared to participants without HIV, were more likely to have received at least one dose of a COVID-19 vaccine (76.2% vs. 62.0%, *p* < 0.001), have a lower BMI (27.3 kg/m^2^ vs. 28.2 kg/m^2^, *p* = 0.004), present with substance use (77.2% vs. 42.9%, *p* < 0.001), and present with comorbidities (such as hypertension, diabetes, autoimmune disease, obesity, and/or CKD) (72.8% vs. 48.2%, *p* < 0.001).

Participants without HIV who had not received a COVID-19 vaccine, compared to vaccinated participants without HIV, were more likely to be younger (53.6 [41.5–60.5] vs. 59.5 [51.1–64.8], *p* < 0.001), Black, non-Hispanic (52.0% vs. 37.9%, *p* < 0.001), and use substances including marijuana (29.8% vs. 15.3%, *p* < 0.001), cocaine (10.2% vs. 4.5%, *p* < 0.001), and tobacco (42.3% vs. 28.1%, *p* < 0.001) (Table 1). Additionally, unvaccinated participants without HIV were more likely to have been diagnosed with a substance use disorder (9.4% vs. 4.7%, *p* = 0.005), but less likely to have been diagnosed with comorbidities including hypertension (33.5% vs. 43.7%, *p* = 0.002) and diabetes (12.8% vs. 21.4%, *p* = 0.001).

PLWH who had not received a COVID-19 vaccine, compared to vaccinated PLWH, were more likely to be Black, non-Hispanic (80.6% vs. 61.6%, *p* = 0.004), and to engage in hazardous drinking (26.7% vs. 14.5%, *p* = 0.006), marijuana use (31.2% vs. 26.6%, *p* = 0.004), and cocaine use (16.1% vs. 10.1%, *p* = 0.004) (Table 1). Additionally, unvaccinated PLWH were less likely to have been diagnosed with comorbidities including diabetes (14.0% vs. 24.6%, *p* = 0.045) and CKD (1.1% vs. 7.1%, *p* = 0.036). However, vaccinated PLWH, compared to unvaccinated PLWH, had a higher median CD4^+^ T cell count (577.5 [416.8–876.8] vs. 517.5 [244.5–722.0], *p* = 0.011) and were more likely to be virally suppressed (76.4% vs. 54.8%, *p* < 0.001).

COVID-19-vaccinated PLWH, compared to vaccinated participants without HIV, were more likely to be male (56.6% vs. 46.0%, *p* < 0.001), Black, non-Hispanic (61.6% vs. 37.9%, *p* < 0.001), have a lower BMI (27.4 [24.0–31.7] vs. 28.3 [25.0–32.9], *p* = 0.04), and use substances, including hazardous alcohol use (14.5% vs. 4.9%, *p* < 0.001), marijuana (26.6% vs. 15.3%, *p* < 0.001), cocaine (10.1% vs. 4.5%, *p* < 0.001), and tobacco (34.7% vs. 28.1%, *p* = 0.047) (Table 1). Vaccinated PLWH were also more likely to have been diagnosed with a substance use disorder (9.4% vs. 4.7%, *p* = 0.006) and comorbidities including hypertension (54.5% vs. 43.7%, *p* = 0.002) and CKD (7.1% vs. 2.3%, *p* < 0.001).

**Figure 1:**
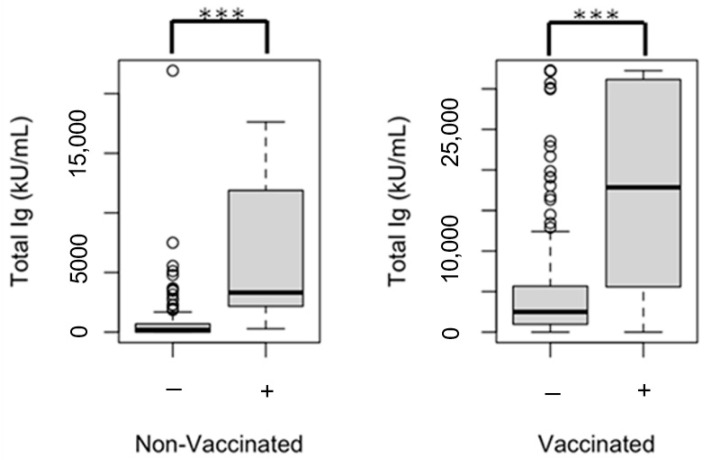
The SARS-CoV-2 spike (trimer) total IgG/IgM/IgA (total Ig) of SARS-CoV-2 nucleocapsid IgG seronegative (nucleocapsid IgG < 20 U/mL; indicated via [−] in figure) and nucleocapsid IgG seropositive (nucleocapsid IgG ≥ 20 U/mL; indicated via [+] in figure) people living with HIV; *** *p* < 0.001.

COVID-19-vaccinated PLWH, compared to vaccinated participants without HIV, were more likely to be SARS-CoV-2 nucleocapsid IgG seropositive (nucleocapsid IgG titer ≥ 20 U/m; 8.1% vs. 3.8%, *p* = 0.008), suggesting prior natural SARS-CoV-2 infection (Table 1). The median SARS-CoV-2 spike (trimer) total Ig titer of PLWH with a prior natural SARS-CoV-2 infection was significantly higher than that in non-exposed PLWH (14,115 [3314–30,704] kU/mL vs. 1740 [395–4590] kU/mL, *p* < 0.001), regardless of vaccination status (Figure 1).

Among COVID-19-vaccinated PLWH, 88.9% received two doses (Table 2). The SARS-CoV-2 spike (trimer) total Ig seropositive rate (Ig ≥ 1000 U/mL; indicates a strong humoral immune response to COVID-19 vaccination) was significantly lower in PLWH who had received only one dose of a COVID-19 vaccine, compared with PLWH who had received two doses (33.3% vs. 81.1%, *p* < 0.001).

**Figure 2:**
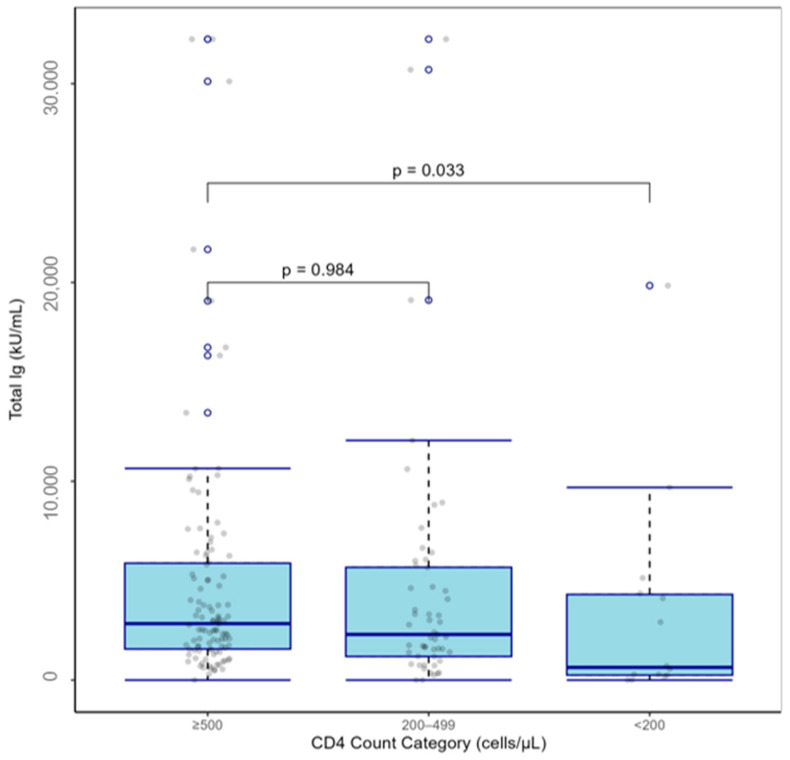
Boxplot of SARS-CoV-2 spike (trimer) total Ig titers by CD4^+^ T cell count category in people living with HIV who received two doses of a COVID-19 vaccine (*n* = 174).

**Figure 3:**
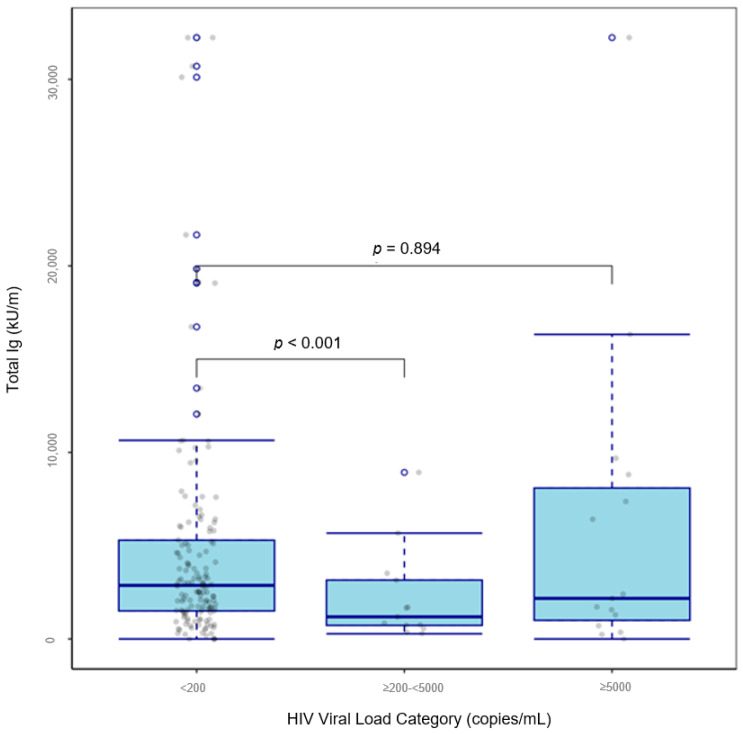
Boxplot of SARS-CoV-2 spike (trimer) total Ig titers by HIV viral load category in people living with HIV who received two doses of a COVID-19 vaccine (*n* = 174).

After applying the exclusion criteria for the main analysis, the regression analysis consisted of 174 PLWH who received two doses of a COVID-19 vaccine (Supplementary Figure S1 and Table S1). SARS-CoV-2 spike (trimer) total Ig seropositive rates among PLWH who received two doses of a COVID-19 vaccine were 42.9% in those with CD4^+^ T cell counts < 200 cells/µL, 77.4% in those with ≥200–<500 cells/µL, and 86.0% in those with ≥500 cells/µL (*p* < 0.001). We also found that median SARS-CoV-2 spike (trimer) total Ig titers were lowest in PLWH with CD4^+^ T cell counts < 200 (*p* = 0.033; Figure 2) and in PLWH with HIV viral loads ≥200–<5000 (*p* < 0.001; Figure 3). There were no differences in SARS-CoV-2 spike (trimer) total Ig titer between obese and non-obese PLWH who received two doses of a COVID-19 vaccine when using the established cut-off of ≥30 kg/m^2^. However, those with a BMI < 27 kg/m^2^ presented with lower SARS-CoV-2 spike (trimer) total Ig titer than those with a BMI ≥ 27 kg/m^2^ (2186 vs. 3007, *p* = 0.057). The adjusted regression model showed that CD4^+^ T cell count (<200 vs. ≥500, β = −0.279, *p* = 0.018), HIV viral load (≥200–<5000 vs. <200, β = −0.35, *p* = 0.002), and days between second vaccination dose date and serology sample collection date (β = −0.003, *p* < 0.001) predicted Box–Cox-transformed SARS-CoV-2 spike (trimer) total Ig titers in PLWH who received two doses of a COVID-19 vaccine (Table 3). A reduced model containing variables associated (*p* ≤ 0.05) with SARS-CoV-2 spike (trimer) total Ig titers in univariate or multivariable regression confirmed that CD4^+^ T cell count (<200 vs. ≥500, β = −0.400, *p* = 0.033), HIV viral load (≥200–<5000 vs. <200, β = −0.275, *p* < 0.001), and days between second vaccination date and serology sample collection date (β = −0.003, *p* < 0.001) significantly predicted SARS-CoV-2 spike (trimer) total Ig titers in PLWH who received two doses of a COVID-19 vaccine (Table 4). A supplementary sensitivity analysis in which we compared the findings from the overall dataset of PLWH who received two doses of a COVID-19 vaccine (*n* = 264) with the subset used in the regression model (*n* = 174), was also conducted and we found that the results from the two analyses were consistent in terms of the direction and significance of key associations (Supplementary Table S2).

## 4. Discussion

Understanding immune response is essential for preparing for public health crises. COVID-19 vaccination effectively provides robust immunity against multiple SARS-CoV-2 variants and reduces severe illness, hospitalization, and mortality [17,18,19,20,21,22]. However, immunocompromised populations may have weaker immune responses to vaccination [24]. Investigations into the immunogenicity of various vaccines in PLWH have reported diminished immune responses [10,11,12,13,14,15,30,31]. At the same time, COVID-19 vaccination has been reported to be just as effective in PLWH on ART with normal CD4^+^ T cell counts and a suppressed viral load, compared to uninfected controls [23,24,25,26,27]. However, other studies have reported diminished responses to COVID-19 vaccination in PLWH with lower CD4^+^ T cell counts [24]. Additionally, vaccine response research, including COVID-19 vaccines, is lacking in racial/ethnic minorities such as Black and Hispanic communities [23,25,26]. This lack of consensus and the scarcity of research in minority populations led us to investigate the immunogenicity of SARS-CoV-2 vaccination in a cohort of underserved Black and Hispanic adults living with and without HIV and high rates of substance use and comorbidities [39], a demographic historically underrepresented in vaccine research [49]. We found that PLWH were more likely to have received a COVID-19 vaccine and present with substance use and comorbidities. Vaccinated PLWH had higher CD4^+^ T cell counts and were more likely to be virally suppressed. A lower CD4^+^ T cell count and higher HIV viral load were associated with lower spike (trimer) total Ig titers in vaccinated PLWH, indicating a diminished response to COVID-19 vaccination.

Compared to participants without HIV, PLWH were more likely to have received a COVID-19 vaccine and present with substance use and comorbidities. Our vaccination rate of 76.2% among PLWH exceeded the national average of 73% in December 2021 [50]. It is possible that PLWH in our cohort were more likely to receive a COVID-19 vaccine due to engagement in care and long-standing rapport with infectious disease care providers. Indeed, we previously reported that MASH cohort PLWH were more likely to engage in preventive measures and healthcare during the COVID-19 pandemic compared to uninfected peers [51].

Compared to COVID-19-vaccinated PLWH, unvaccinated PLWH were more likely to be Black non-Hispanic, and engage in hazardous drinking, marijuana use, and cocaine use, but less likely to have been diagnosed with comorbidities including diabetes and CKD. These findings concur with previous research that reported lower COVID-19 vaccine rates and greater vaccine hesitancy in Black non-Hispanic populations, likely due to socioeconomic disparities and other challenges [52,53,54]. We also previously reported on the relationship between substance use and lower odds of COVID-19 vaccination and greater vaccine hesitancy in the MASH and RADx-UP cohorts [40]. Our findings regarding unvaccinated PLWH being less likely to present with comorbidities concur with previous work reporting increased vaccine acceptance among those with a higher risk of severe outcomes [55]. Thus, PLWH with better health and fewer comorbidities may have not received a COVID-19 vaccine due to a lower perceived need or susceptibility.

CD4^+^ T cell counts are heavily influenced by ART initiation and adherence [56,57,58]. While we found similar ART prescription rates between COVID-19-vaccinated PLWH and unvaccinated PLWH, vaccinated PLWH had higher CD4^+^ T cell counts and were more likely to be virally suppressed. This suggests that vaccine acceptance may correlate with better ART adherence, despite the similar rates of ART prescription. Given that participants were recruited through HIV care settings, the lower viral suppression rates among unvaccinated PLWH, particularly those with fewer comorbidities and more substance use disorders, likely reflects poorer engagement with care and medication adherence, resulting in both suboptimal HIV control and lower vaccine uptake.

Among vaccinated PLWH, SARS-CoV-2 spike (trimer) total Ig seropositive rates increased along with CD4^+^ T cell count and were the highest among PLWH with ≥500 cells. A lower CD4^+^ T cell count and higher HIV viral load were associated with lower total SARS-CoV-2 spike (trimer) total Ig titers, indicating a diminished response to COVID-19 vaccination. A similar study conducted in 2021 reported significantly lower SARS-CoV-2 anti-spike antibody titers among PLWH with CD4^+^ T cell counts of <500 cells, and, notably, <200 cells, following two-dose COVID-19 vaccination [28]. Nault et al. also reported a significant relationship between CD4^+^ T cell count and anti-receptor-binding domain (RBD) IgG response, which was lowest in PLWH with CD4^+^ T cell counts < 250 cells [24]. Thus, our work confirms a positive association between CD4^+^ T cell count and COVID-19 vaccine immune response, but in a sample of Black and Hispanic adults, a population lacking in COVID-19 vaccine research [23,25,26]. Our findings are also novel in that in addition to lower CD4^+^ T cell count, we also demonstrated a relationship between higher HIV viral load and diminished COVID-19 vaccination response, which was not previously reported [59].

Our findings could be explained by the notion that CD4^+^ T cells, the target of HIV [24], play a crucial role in orchestrating immune response through coordinating and regulating B cells involved in antibody (Ig) production [24,60,61,62]. Although the improvements in and increased access to ART has made immune recovery possible, subtle defects in inflammation and immune function persist, which may impair vaccine response [24]. Thus, in the future, it may be recommended that vaccination strategies in PLWH be tailored to CD4^+^ T cell count, as the functionality of B cells, and consequently, antibody (Ig) production, relies on CD4^+^ T cells [63]. In PLWH with low CD4^+^ T cell counts, additional vaccination doses may be advised to produce antibody titers sufficient to promote long-lasting immunity. Indeed, the SARS-CoV-2 spike (trimer) total Ig seropositive rate was significantly lower in PLWH who had received only one dose of a COVID-19 vaccine compared with PLWH who had received two doses, reinforcing CDC guidelines that include additional doses and more frequent boosters [16,26].

This work is strengthened by the use of a large sample of underserved minority adults from Miami, Florida, which experiences a high level of social vulnerability [64], the inclusion of similar proportions of men and women living with and without HIV and COVID-19 vaccination, the representation of Black and Hispanic adults, a population historically underrepresented in vaccine research [23,25,26,49], and the adequate distribution of CD4^+^ T cell counts ranging from <200 to >500, allowing for proper stratification and regression analyses. We also conducted a sensitivity analysis in which we compared the findings from the overall dataset of PLWH who received two doses of a COVID-19 vaccine (*n* = 264) with the subset used in the regression model (*n* = 174) and found that the results from the two analyses were consistent in terms of the direction and significance of key associations. Limitations include the cross-sectional design, there being no information on nadir CD4^+^ T cell counts, and the fact that a comparison between different types of COVID-19 vaccines was not performed due to the small sample size of participants who did not receive the mRNA-1273 or BNT162b2 mRNA COVID-19 vaccines. Additionally, because data were collected during a time when CDC COVID-19 vaccination guidelines were changing rapidly, complete booster and additional dose data were not yet available. The majority of the cohort, both PLWH and HIV-uninfected participants, received a COVID-19 vaccine, which limits our ability to draw conclusions regarding unvaccinated participants. Further, although we excluded nucleocapsid IgG seropositive cases from regression analysis to rule out prior natural infection, the half-life of nucleocapsid antibodies is relatively short [65]. Thus, it is possible some cases of prior natural SARS-CoV-2 infection were not identified. Finally, the final reduced model had an adjusted R^2^ = 0.156, which suggests modest explanatory power. However, we emphasize that our primary goal was to identify statistically significant associations rather than to maximize the explained variance. Considering this, we caution readers to consider these aspects when interpreting the scope and generalizability of our conclusions.

In conclusion, PLWH were more likely to receive a COVID-19 vaccine, and vaccinated PLWH had higher CD4^+^ T cell counts and were more likely to be virally suppressed. A lower CD4^+^ T cell count and higher HIV viral load were associated with a lower SARS-CoV-2 spike (trimer) total Ig titers in COVID-19-vaccinated PLWH. Thus, we provide evidence of a relationship between lower CD4^+^ T cell count and higher HIV viremia with reduced SARS-CoV-2 immunogenicity in racial/ethnic minority men and women living with HIV. HIV care providers should target efforts to maintain viral suppression to optimize immune responses to COVID-19 vaccination and may consider booster doses or modified dosing in PLWH [24], depending on immunosuppression level.

## Figures and Tables

**Table 1:** RADx-UP sample characteristics according to HIV serostatus and COVID-19 vaccination status (*n* = 1317).

	People Without HIV (*n* = 927)			PLWH (*n* = 390)			*p* ^k^
Variable ^a^	Non-Vaccinated (*n* = 352)	Vaccinated (*n* = 575, 62.0%)	*p*	Non-Vaccinated (*n* = 93)	Vaccinated (*n* = 297, 76.2%)	*p*	
Sex, male	169 (48.0)	265 (46.0)	0.569	54 (58.1)	168 (56.6)	0.893	<0.001
Age, years	53.6 (41.5–60.5)	59.5 (51.1–64.8)	<0.001	56.2 (52.5–63.0)	58.4 (54.8–63.7)	0.102	0.075
Race/Ethnicity							
Black, non-Hispanic	183 (52.0)	218 (37.9)	<0.001	75 (80.6)	183 (61.6)	0.004	<0.001
White, non-Hispanic	22 (6.3)	25 (4.3)		3 (3.2)	19 (6.4)		
White, Hispanic	120 (34.1)	291 (50.6)		11 (11.8)	82 (27.6)		
Other ^b^	27 (7.7)	41 (7.1)		4 (4.3)	13 (4.4)		
CD4^+^ T, (cells/µL)	N/A	N/A	N/A	517.5 (244.5–722.0)	577.5 (416.8–876.8)	0.011	N/A
<200				17 (18.2)	23 (7.7)	0.010	N/A
≥200–<500				21 (22.6)	81 (27.2)		
≥500				40 (43.0)	160 (53.9)		
Unknown/missing				15 (16.1)	33 (11.1)		
On ART	N/A	N/A	N/A	89 (95.7)	275 (92.6)	0.770	N/A
HIV viral load, (copies/mL)	N/A	N/A	N/A				
<200				51 (54.8)	227 (76.4)	<0.001	N/A
≥200–<5000				10 (10.8)	17 (5.7)		
>5000				13 (14.0)	19 (6.4)		
Unknown/missing				19 (20.4)	34 (11.4)		
Log_(10)_ HIV viral load (copies/mL)				1.24 (0.70–3.27)	1.17 (0.70–1.76)	0.004	N/A
BMI, kg/m^2^	27.7 (24.4–33.0)	28.3 (25.0–32.9)	0.928	25.8 (23.1–30.2)	27.4 (24.0–31.7)	0.054	0.040
<18.5	2 (0.6)	5 (0.9)	0.610	3 (3.2)	8 (2.7)	0.163	0.046
≥18.5–<25	103 (29.3)	138 (24.0)		37 (39.8)	84 (28.2)		
≥25–<30	119 (33.8)	211 (36.7)		29 (31.2)	102 (34.2)		
≥30	128 (36.4)	221 (38.4)		24 (25.8)	103 (34.6)		
Interval between vaccination dose 2 and serology collection, days	N/A	100.0 (65.0–133.3)	N/A	N/A	101.4 (56.0–127.0)	N/A	0.051
<14		27 (4.7)			17 (5.7)		<0.001
≥14–<180		415 (72.2)			243 (81.8)		
≥180		133 (23.1)			37 (12.5)		
Substance use	174 (49.4)	225 (39.1)	0.002	70 (75.3)	232 (78.1)	0.567	<0.001
Hazardous drinking ^c^	31 (8.8)	28 (4.9)	0.972	25 (26.7)	43 (14.5)	0.006	<0.001
Drug use, any ^d^	118 (33.5)	101 (17.5)	<0.001	35 (37.6)	94 (31.6)	0.345	<0.001
Marijuana	105 (29.8)	88 (15.3)	<0.001	29 (31.2)	79 (26.6)	0.004	<0.001
Cocaine	36 (10.2)	26 (4.5)		15 (16.1)	30 (10.1)		
Other drugs ^e^	4 (1.1)	4 (0.7)		3 (3.3)	6 (2.4)		
Cigarette smoking	149 (42.3)	162 (28.1)	<0.001	41 (44.1)	103 (34.7)	0.101	0.047
Substance use disorder	33 (9.4)	27 (4.7)	0.005	11 (11.8)	28 (9.4)	0.501	0.006
Comorbidities							
Hypertension	118 (33.5)	251 (43.7)	0.002	45 (48.4)	162 (54.5)	0.358	0.002
Diabetes	45 (12.8)	123 (21.4)	0.001	13 (14.0)	73 (24.6)	0.045	0.286
Autoimmune disease	7 (2.0)	21 (3.7)	0.159	4 (4.3)	16 (5.4)	0.794	0.231
Obesity	128 (36.4)	221 (38.4)	0.248	24 (25.8)	103 (34.7)	0.142	<0.001
Chronic kidney disease	3 (0.9)	13 (2.3)	0.124	1 (1.1)	21 (7.1)	0.036	<0.001
≥Comorbidity ^f^	199 (56.5)	378 (65.7)	0.015	59 (63.4)	225 (75.8)	0.028	0.002
SARS-CoV-2 spike (trimer) total Ig, (kU/mL) ^g^	473 (0–2251)	2927 (1114–6411)	<0.001	209 (0–1353)	2786 (1049–6415)	<0.001	0.650
≥1000 ^h^	125 (35.5)	446 (77.6)	<0.001	25 (26.9)	207 (74.2)	<0.001	0.453
SARS-CoV-2 nucleocapsid IgG, (U/mL) ^i^	2.26 (1.34–4.36)	2.16 (1.19–4.16)	0.001	2.12 (1.38–4.75)	2.40 (1.20–6.72)	0.698	0.044
≥20 ^j^	33 (9.4)	22 (3.8)	0.001	5 (5.4)	24 (8.1)	0.522	0.008

^a^ Data are presented as count (percent, %) and median (interquartile range) for continuous variables. ^b^ Includes Black Hispanic, American Indian or Alaska Native, Asian, Native Hawaiian or other Pacific Islander, mixed-race, and some other race not captured by response options. ^c^ Based on the National Institute of Alcohol Abuse and Alcoholism guidelines. ^d^ Use of marijuana, cocaine/crack, heroin, fentanyl, methamphetamine, amphetamine, hallucinogens, ecstasy, or misuse of prescription drugs in the past 12 months. ^e^ Use of heroin, fentanyl, methamphetamine, amphetamine, hallucinogens, or ecstasy in the past 12 months. ^f^ Participants reporting at least one of the following comorbidities: hypertension, diabetes, autoimmune disease, obesity, and/or chronic kidney disease. ^g^ SARS-CoV-2 spike (trimer) total IgG/IgM/IgA, indicates immune response to COVID-19 vaccination. ^h^ Total Ig above 1000 kU/mL is considered seropositive against the SARS-CoV-2 spike. ^i^ SARS-CoV-2 nucleocapsid IgG, indicates prior natural SARS-CoV-2 infection. ^j^ SARS-CoV-2 nucleocapsid IgG ≥20 U/mL suggests prior natural SARS-CoV-2 infection. ^k^ Difference between COVID-19 vaccinated HIV-uninfected participants and COVID-19-vaccinated PLWH. Abbreviations: BMI, body mass index; PLWH, people living with HIV; RADx-UP, Rapid Acceleration of Diagnostics-Underserved Populations.

**Table 2:** RADx-UP sample characteristics of COVID-19-vaccinated PLWH according to number of COVID-19 vaccine doses (*n* = 297).

Variable ^a^	1 Dose (*n* = 33)	2 Doses (*n* = 264, 88.9%)	*p*
Sex, male	22 (66.7)	146 (55.3)	0.214
Age, years	56.2 (52.8–60.1)	58.9 (55.2–63.9)	0.647
Race/Ethnicity			0.843
Black, non-Hispanic	18 (54.6)	165 (62.5)	
White, non-Hispanic	3 (9.1)	16 (6.1)	
White, Hispanic	12 (36.4)	70 (26.5)	
Other ^b^	0 (0)	13 (4.9)	
Vaccine brand			N/A ^k^
Ad26.COV2.S COVID-19 vaccine	22 (66.7)	3 (1.1)	
mRNA-1273 or BNT162b2 mRNA COVID-19 vaccine	11 (33.3)	253 (95.8)	
Other	0 (0)	8 (3.0)	
CD4^+^ T, (cells/µL)	554.0 (385.0–743.5)	581.0 (419.0–886.0)	0.488
<200	5 (15.2)	18 (6.0)	0.523
≥200–<500	8 (24.2)	73 (24.5)	
≥500	18 (54.5)	142 (47.7)	
Unknown/missing	2 (6.1)	32 (10.7)	
On ART	30 (90.9)	245 (92.8)	
HIV viral load, (copies/mL)			
<200	27 (81.8)	200 (75.8)	0.900
≥200–<5000	1 (3.0)	16 (6.1)	
>5000	3 (9.1)	16 (6.1)	
Unknown/missing	2 (6.1)	33 (12.5)	
Log_(10)_ HIV viral load (copies/mL)	1.18 (0.7–1.98)	1.18 (0.69–1.71)	0.805
BMI, kg/m^2^	27.0 (23.1–31.6)	27.5 (24.2–31.8)	0.584
<18.5	1 (3.0)	7 (2.7)	0.991
≥18.5–<25	11 (33.3)	73 (27.7)	
≥25–<30	10 (30.3)	92 (34.8)	
≥30	11 (33.3)	92 (34.8)	
Interval between vaccination dose 2 and serology collection, days	81.0 (50.0–104.0)	88.5 (56.0–136.0)	0.887
<14	6 (18.2)	11 (4.2)	0.711
≥14–<180	26 (78.8)	217 (82.2)	
≥180	1 (3.0)	36 (13.6)	
Substance use	26 (78.8)	206 (78.0)	0.860
Hazardous drinking ^c^	4 (12.1)	41 (15.5)	0.206
Drug use, any ^d^	15 (45.5)	79 (29.9)	0.059
Marijuana	14 (42.4)	65 (24.6)	0.086
Cocaine	5 (15.2)	25 (9.5)	0.188
Other drugs ^e^	2 (6.1)	5 (1.9)	0.980
Cigarette smoking	17 (51.5)	86 (32.6)	0.275
Substance use disorder	3 (9.1)	25 (9.5)	0.540
Comorbidities			
Hypertension	16 (48.5)	146 (55.3)	0.970
Diabetes	7 (21.2)	66 (25.0)	0.456
Autoimmune disease	1 (3.0)	15 (5.7)	0.630
Obesity	11 (33.3)	92 (34.8)	0.532
Chronic kidney disease	1 (3.0)	20 (7.6)	0.266
≥comorbidity ^f^	23 (69.7)	202 (76.5)	0.577
SARS-CoV-2 spike (trimer) total Ig, (kU/mL) ^g^	610 (72–2553)	2949 (1404–6718)	0.048
≥1000 ^h^	11 (33.3)	214 (81.1)	<0.001
SARS-CoV-2 nucleocapsid IgG, (U/mL) ^i^	1.73 (0.87–3.54)	2.44 (1.24–7.24)	0.700
≥20 U/mL ^j^	2 (6.1)	22 (8.3)	0.524

^a^ Data are presented as count (percent, %) and median (interquartile range) for continuous variables. ^b^ Includes Black Hispanic, American Indian or Alaska Native, Asian, Native Hawaiian or other Pacific Islander, mixed-race, and some other race not captured by response options. ^c^ Based on the National Institute of Alcohol Abuse and Alcoholism guidelines. ^d^ Use of marijuana, cocaine/crack, heroin, fentanyl, methamphetamine, amphetamine, hallucinogens, ecstasy, or misuse of prescription drugs in the past 12 months. ^e^ Use of heroin, fentanyl, methamphetamine, amphetamine, hallucinogens, or ecstasy in the past 12 months. ^f^ Participants reporting at least one of the following comorbidities: hypertension, diabetes, autoimmune disease, obesity, and/or chronic kidney disease. ^g^ SARS-CoV-2 spike (trimer) total IgG/IgM/IgA, indicates immune response to COVID-19 vaccination. ^h^ Total Ig above 1000 kU/mL is considered seropositive against the SARS-CoV-2 spike. ^i^ SARS-CoV-2 nucleocapsid IgG indicates prior natural SARS-CoV-2 infection. ^j^ SARS-CoV-2 nucleocapsid IgG ≥ 20 U/mL suggests prior natural SARS-CoV-2 infection. ^k^ Unable to calculate *p*-value due to small cell counts. Abbreviations: BMI, body mass index; PLWH, people living with HIV; RADx-UP, Rapid Acceleration of Diagnostics-Underserved Populations.

**Table 3:** Univariate and multivariable linear regression models for Box–Cox-transformed SARS-CoV-2 spike (trimer) total Ig titers in PLWH who received two doses of a COVID-19 vaccine (*n* = 174).

Variable	Adjusted Model			Unadjusted Model		
	β [95% CI]	t	*p*	β	t	*p*
Sex						
Female	−0.072 [−0.232, 0.088]	−1.162	0.247	0.038	0.655	0.513
Male	Reference			Reference		
Age, years	−0.044 [−0.214, 0.126]	−0.351	0.726	0.031	0.338	0.736
<50	−0.044 [−0.164, 0.076]	−0.351	0.726	0.031	0.338	0.736
≥50–<55	−0.083 [−0.193, 0.027]	−0.775	0.440	0.052	0.612	0.541
≥55–<60	−0.124 [−0.274, 0.026]	−1.422	0.157	0.016	0.15	0.881
≥60–<65	−0.026 [−0.126, 0.074]	−0.275	0.783	0.117	0.948	0.344
≥65	Reference			Reference		
Race/Ethnicity						
White, non-Hispanic	−0.123 [−0.237, −0.101]	−1.041	0.300	−0.062	−0.517	0.606
White, Hispanic	0.064 [0.058, 0.069]	0.877	0.382	−0.015	−0.203	0.839
Other ^a^	−0.163 [−0.343, 0.017]	−1.186	0.238	0.011	0.079	0.937
Black, non-Hispanic	Reference			Reference		
CD4^+^ T, (cells/µL)						
<200	−0.279 [−0.439, −0.119]	−2.394	0.018	−0.304	−2.692	0.008
≥200–<500	0.004 [0.003, 0.006]	0.053	0.958	−0.059	−0.938	0.35
≥500	Reference			Reference		
HIV viral load, (copies/mL)						
≥200–<5000	−0.35 [−0.45, −0.25]	−3.1	0.002	−0.271	−2.52	0.01
≥5000	−0.009 [−0.099, 0.081]	−0.079	0.937	−0.024	−0.232	0.817
<200	Reference			Reference		
BMI, kg/m^2^						
≥27	0.151 [0.144, 0.158]	1.907	0.058	0.151	2.635	0.009
<27	Reference			Reference		
Interval between vaccination dose 2 and serology collection, days	−0.003 [−0.153, 0.147]	−4.228	<0.001	−0.003	−3.488	<0.001
Substance use						
Hazardous drinking ^b^	0.060 [−0.05, 0.17]	0.816	0.416	0.033	0.432	0.666
Marijuana	−0.023 [−0.223, 0.177]	−0.321	0.215	−0.077	−1.172	0.243
Cocaine	−0.132 [−0.272, 0.008]	−1.245	0.544	−0.195	−1.933	0.055
Other drugs ^c^	−0.166 [−0.356, 0.024]	−0.609	0.469	−0.051	−0.192	0.848
Cigarette smoking	0.046 [−0.124, 0.216]	0.726	0.397	−0.01	−0.161	0.872
Substance use disorder	−0.09 [−0.25, 0.07]	−0.85	0.400	−0.085	−0.857	0.393
Comorbidities						
Hypertension	−0.121 [−0.301, 0.059]	−1.906	0.059	−0.092	−1.591	0.113
Diabetes	0.129 [−0.081, 0.339]	1.937	0.055	0.101	1.52	0.130
Autoimmune disease	0.013 [−0.097, 0.123]	0.11	0.912	0.072	0.616	0.539
Obesity	0.011 [−0.099, 0.121]	0.13	0.896	0.106	1.792	0.075
Chronic kidney disease	0.029 [−0.141, 0.199]	0.279	0.781	−0.091	−0.89	0.375
≥Comorbidity ^d^	−0.06 [−0.28, 0.16]	−0.819	0.414	−0.042	−0.602	0.548

^a^ Includes Black Hispanic, American Indian or Alaska Native, Asian, Native Hawaiian or other Pacific Islander, mixed-race, and some other race not captured by response options. ^b^ Based on the National Institute of Alcohol Abuse and Alcoholism guidelines. ^c^ Use of heroin, fentanyl, methamphetamine, amphetamine, hallucinogens, or ecstasy in the past 12 months. ^d^ Participants reporting at least one of the following comorbidities: hypertension, diabetes, autoimmune disease, obesity, and/or chronic kidney disease. Abbreviations: BMI, body mass index; CI, confidence interval; PLWH, people living with HIV.

**Table 4:** Final reduced model for Box–Cox-transformed SARS-CoV-2 spike (trimer) total Ig titers in PLWH who received two doses of a COVID-19 vaccine (*n* = 174).

Variables	Adjusted Model		
	β [95%CI]	t	*p*
CD4^+^ T, (cells/µL)			
<200	−0.400 [−0.59, −0.21]	−2.151	0.033
≥200–<500	0.001 [−0.109, 0.111]	0.02	0.984
≥500	Reference		
HIV viral load, (copies/mL)			
≥200–<5000	−0.275 [−0.485, −0.065]	−2.654	<0.001
≥5000	0.0135 [−0.117, 0.144]	0.133	0.894
<200	Reference		
BMI, (kg/m^2^)			
≥27	0.107 [−0.043, 0.257]	1.74	0.050
<27	Reference		
Interval between vaccination dose 2 and serology collection, days	−0.003 [−0.247, 0.253]	3.764	<0.001
Substance use			
Cocaine	−0.15 [−0.34, 0.04]	0.545	0.124
Comorbidities			
Hypertension	−0.08 [−0.22, 0.06]	−1.427	0.156
Diabetes	0.117 [0.07, 0.227]	1.868	0.060

Adjusted R^2^ = 0.156; F-statistic = 5.438 on 7 and 161 degrees of freedom; value = 1.281 × 10 ^−5^. Abbreviations: BMI, body mass index; CI, confidence interval; PLWH, people living with HIV.

## Data Availability

The data presented in this study are available on request from the corresponding author. The data are not publicly available due to privacy and confidentiality restrictions. Requests to access the Rapid Acceleration of Diagnostics-Underserved Populations (RADx-UP) common data elements (CDES) datasets should be directed to the RADx-UP Data Core, at radx-up-cdcc@dm.duke.edu.

## Supplementary Materials

The following supporting information can be downloaded at: https://www.mdpi.com/article/10.3390/vaccines13050517/s1, Figure S1. Flow diagram of participant recruitment, eligibility, and final inclusion into multivariable regression analysis. Table S1. Characteristics of RADx-UP PLWH who received two doses of a COVID-19 vaccine included in the multivariable linear regression models for Box-Cox-transformed SARS-CoV-2 spike (trimer) total Ig titers (*n* = 174). Table S2. Evaluation of MAR assumption: Comparing datasets of PLWH with two doses of a COVID-19 vaccine (*n* = 264) to PLWH with two doses of a COVID-19 vaccine included in the final multivariable regression model (*n* = 174).
