# Supplementary material for: SARS-CoV-2 Antibodies in Response to COVID-19 Vaccination in Underserved Racial/Ethnic Minority People Living with HIV
Source: Vaccines (Basel). 2025 May 13;13(5):517. doi: 10.3390/vaccines13050517 (PMC12116134; doi:10.3390/vaccines13050517)
Supplement: Supplementary file 1 [file vaccines-13-00517-s001.zip › vaccines-3529831-supplementary.pdf]

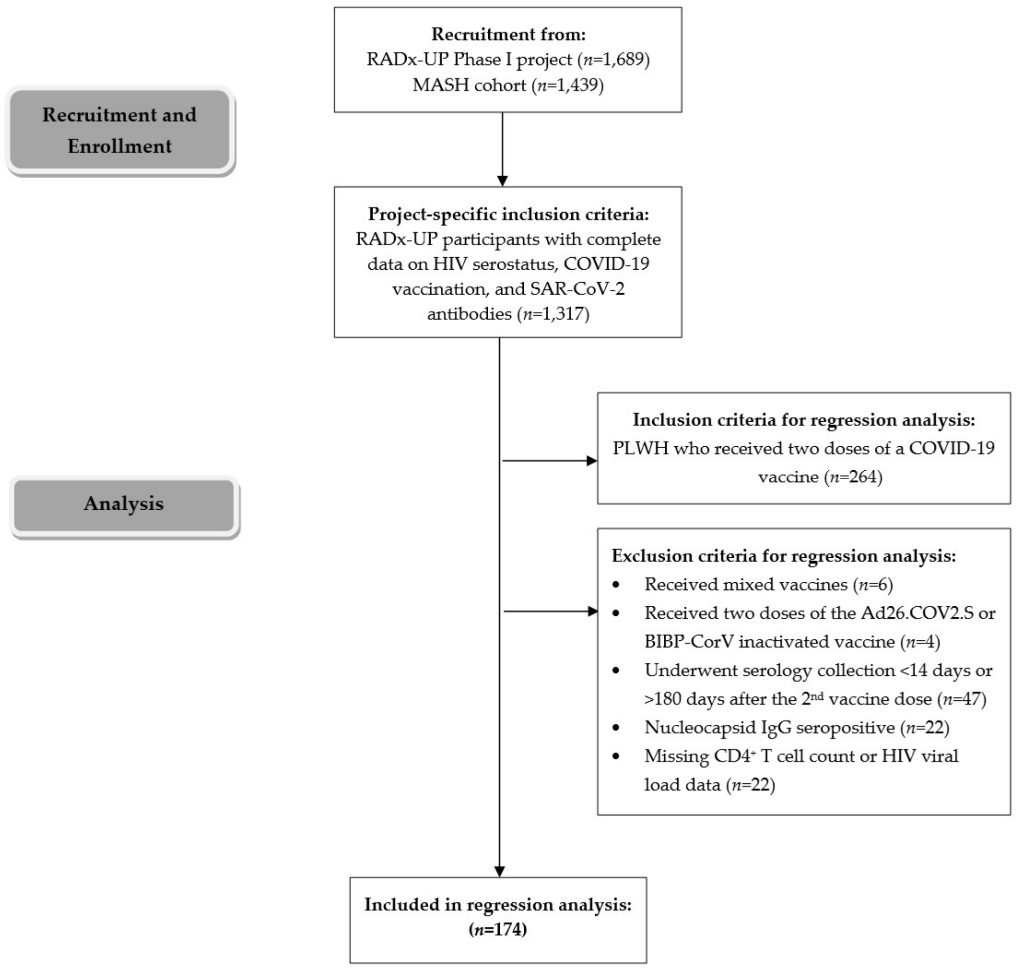


**Supplementary Figure S1.** Flow diagram of participant recruitment, eligibility, and final inclusion into multivariable regression analysis.

**Supplementary Table S1**. Characteristics of RADx-UP PLWH who received two doses of a COVID-19 vaccine included in the multivariable linear regression models for Box-Cox-transformed SARS-CoV-2 spike (trimer) total Ig titers ($n$ = 174).

| Variable [a] | | |
|---|---|---|
| **Sex** | | *n*=174 |
| | Female | 80 (46) |
| | Male | 94 (54) |
| **Age**, years | | |
| | <50 | 14 (8) |
| | ≥50-<55 | 21 (12.1) |
| | ≥55-<60 | 66 (37.9) |
| | ≥60-<65 | 43 (24.7) |
| | ≥65 | 30 (17.2) |
| **Race/Ethnicity** | | |
| | White, non-Hispanic | 12 (6.9) |
| | White, Hispanic | 37 (21.3) |
| | Other [b] | 8 (4.6) |
| | Black, non-Hispanic | 117 (67.2) |
| **CD4+ T**, (cells/μL) | | |
| | <200 | 14 (8) |
| | ≥200-<500 | 53 (30.5) |
| | ≥500 | 107 (61.5) |
| **HIV viral load**, (copies/mL) | | |
| | ≥200-<5,000 | 15 (8.6) |
| | ≥5,000 | 13 (7.5) |
| | <200 | 146 (83.9) |
| **BMI**, kg/m$^2$ | | |
| | ≥27 | 96 (55.2) |
| | <27 | 78 (44.8) |
| **Interval between vaccination dose 2 and serology collection**, days | | 85.7 [56-114] |
| **Substance use** | | |
| | Hazardous drinking [c] | 30 (17.2) |
| | Marijuana | 45 (25.9) |
| | Cocaine | 16 (9.2) |
| | Other drugs [d] | 2 (1.1) |
| | Cigarette smoking | 59 (33.9) |
| | Substance use disorder | 16 (9.2) |
| **Comorbidities** | | |
| | Hypertension | 96 (55.2) |
| | Diabetes | 45 (25.9) |
| | Autoimmune disease | 11 (6.3) |
| | Obesity | 66 (37.9) |
| | Chronic kidney disease | 15 (8.6) |
| | ≥Comorbidity [e] | 134 (77) |

a. Data are presented as count (percent, %) and median (interquartile range) for continuous variables.
b. Includes Black Hispanic, American Indian or Alaska Native, Asian, Native Hawaiian or other Pacific Islander, mixed-race, and some other race not captured by response options.

c. Based on the National Institute of Alcohol Abuse and Alcoholism guidelines.
d. Use of heroin, fentanyl, methamphetamine, amphetamine, hallucinogens, or ecstasy in the past 12 months.
e. Participants reporting at least one of the following comorbidities: hypertension, diabetes, autoimmune disease, obesity, and/or chronic kidney disease.

Abbreviations: BMI, body mass index; PLWH, people living with HIV; RADx-UP, Rapid Acceleration of Diagnostics-Underserved Populations

**Supplementary Table S2.** Evaluation of MAR assumption: Comparing datasets of PLWH with two doses of a COVID-19 vaccine (*n* = 264) to PLWH with two doses of a COVID-19 vaccine included in the final multivariable regression model (*n* = 174).

| Variable | Adjusted model (*n*=174) | | | Adjusted model (*n*=264) | | |
|---|---|---|---|---|---|---|
| | *β* | *t* | *p* | *β* | *t* | *p* |
| **Sex** | | | | | | |
| Female | -0.072 | -1.162 | 0.247 | -0.015 | -0.253 | 0.8003 |
| Male | | Reference | | | Reference | |
| **Age**, years | | | | | | |
| <50 | -0.044 | -0.351 | 0.726 | -0.040 | -0.446 | 0.656 |
| ≥50-<55 | -0.083 | -0.775 | 0.440 | -0.055 | -0.068 | 0.946 |
| ≥55-<60 | -0.124 | -1.422 | 0.157 | 0.073 | 0.768 | 0.443 |
| ≥60-<65 | -0.026 | -0.275 | 0.783 | 0.0375 | 0.347 | 0.729 |
| ≥65 | | Reference | | | Reference | |
| **Race/Ethnicity** | | | | | | |
| White, non-Hispanic | -0.123 | -1.041 | 0.300 | -0.116 | -0.940 | 0.349 |
| White, Hispanic | 0.064 | 0.877 | 0.382 | 0.102 | 1.551 | 0.123 |
| Other [a] | -0.163 | -1.186 | 0.238 | 0.147 | 1.252 | 0.212 |
| Black, non-Hispanic | | Reference | | | Reference | |
| **CD4+ T**, (cells/μL) | | | | | | |
| <200 | -0.279 | -2.394 | **0.018** | -0.286 | -2.559 | **0.011** |
| ≥200-<500 | 0.004 | 0.053 | 0.958 | -0.012 | -0.198 | 0.843 |
| ≥500 | | Reference | | | Reference | |
| **HIV viral load**, (copies/mL) | | | | | | |
| ≥200-<5,000 | -0.35 | -3.1 | **0.002** | -0.210 | -1.917 | **0.056** |
| ≥5,000 | -0.009 | -0.079 | 0.937 | -0.020 | -0.173 | 0.863 |
| <200 | | Reference | | | Reference | |
| **BMI**, kg/m$^2$ | | | | | | |
| ≥27 | 0.151 | 1.907 | 0.058 | 0.014 | 0.207 | 0.836 |
| <27 | | Reference | | | Reference | |
| **Interval between vaccination dose 2 and serology collection**, days | -0.003 | -4.228 | **<0.001** | -0.001 | -2.006 | **0.0462** |
| **Substance use** | | | | | | |
| Hazardous drinking [b] | 0.060 | 0.816 | 0.416 | 0.018 | 0.240 | 0.811 |
| Marijuana | -0.023 | -0.321 | 0.215 | -0.069 | -0.979 | 0.329 |
| Cocaine | -0.132 | -1.245 | 0.544 | -0.086 | -0.827 | 0.409 |
| Other drugs [c] | -0.166 | -0.609 | 0.469 | -0.194 | -0.798 | 0.426 |
| Cigarette smoking | 0.046 | 0.726 | 0.397 | -0.004 | -0.067 | 0.947 |
| Substance use disorder | -0.09 | -0.85 | 0.400 | -0.118 | -1.204 | 0.230 |
| **Comorbidities** | | | | | | |
| Hypertension | -0.121 | -1.906 | 0.059 | -0.036 | -0.608 | 0.544 |

| | | | | | | |
|---|---|---|---|---|---|---|
| Diabetes | 0.129 | 1.937 | 0.055 | 0.098 | 1.520 | 0.130 |
| Autoimmune disease | 0.013 | 0.11 | 0.912 | 0.036 | 0.333 | 0.740 |
| Obesity | 0.011 | 0.13 | 0.896 | 0.100 | 1.494 | 0.137 |
| Chronic kidney disease | 0.029 | 0.279 | 0.781 | 0.028 | 0.281 | 0.779 |
| ≥Comorbidity [d] | -0.06 | -0.819 | 0.414 | 0.016 | 0.235 | 0.814 |

a. Includes Black Hispanic, American Indian or Alaska Native, Asian, Native Hawaiian or other Pacific Islander, mixed-race, and some other race not captured by response options.
b. Based on the National Institute of Alcohol Abuse and Alcoholism guidelines.
c. Use of heroin, fentanyl, methamphetamine, amphetamine, hallucinogens, or ecstasy in the past 12 months.
d. Participants reporting at least one of the following comorbidities: hypertension, diabetes, autoimmune disease, obesity, and/or chronic kidney disease.

Abbreviations: BMI, body mass index; MAR, missing at random; PLWH, people living with HIV
